# Comprehensive Assessment of Context-Adaptive Street Lighting: Technical Aspects, Economic Insights, and Measurements from Large-Scale, Long-Term Implementations

**DOI:** 10.3390/s24185942

**Published:** 2024-09-13

**Authors:** Gianni Pasolini, Paolo Toppan, Andrea Toppan, Rudy Bandiera, Mirko Mirabella, Flavio Zabini, Diego Bonata, Oreste Andrisano

**Affiliations:** 1 Department of Electrical, Electronic and Information Engineering “G. Marconi”, University of Bologna, 40126 Bologna, Italy; 2 Wireless Communications Laboratory (WiLab) of the National Inter-University Consortium for Telecommunications (CNIT), 40133 Bologna, Italy; 3 Wi4B s.r.l., 40127 Bologna, Italy; 4 Department of Architecture, University of Bologna, 40126 Bologna, Italy; mirko.mirabella@unibo.it; 5 AstroLight Studio, 24053 Brignano Gera d’Adda, Italy

**Keywords:** smart lighting, street lighting, adaptive dimming, environmental sensing, smart city

## Abstract

This paper addresses the growing importance of efficient street lighting management, driven by rising electricity costs and the need for municipalities to implement cost-effective solutions. Central to this study is the UNI 11248 Italian regulation, which extends the European EN 13201-1 standard introduced in 2016. These standards provide guidelines for designing, installing, operating, and maintaining lighting systems in pedestrian and vehicular traffic areas. Specifically, the UNI 11248 standard introduces the possibility to dynamically adjust light intensity through two alternative operating modes: (a) Traffic Adaptive Installation (TAI), which dims the light based solely on real-time traffic flow measurements; and (b) Full Adaptive Installation (FAI), which, in addition to traffic measurements, also requires evaluating road surface luminance and meteorological conditions. In this paper, we first present the general architecture and operation of an FAI-enabled lighting infrastructure, which relies on environmental sensors and a heterogeneous wireless communication network to connect intelligent, remotely controlled streetlights. Subsequently, we examine large-scale, in-field FAI infrastructures deployed in Vietnam and Italy as case studies, providing substantial measurement data. The paper offers insights into the measured energy consumption of these infrastructures, comparing them to that of conventional light-control strategies used in traditional installations. The measurements demonstrate the superiority of FAI as the most efficient solution.

## 1. Introduction

In 2022, the EU’s total net electricity generation amounted to 2701 Terawatt hours (TWh). A significant portion, 56.1%, originated from non-combustible primary sources, mainly nuclear (21.4%), wind (15.4%), hydro (11.2%), and solar plants (7.7%). The remaining 43.9% was derived from combustible fuels, such as natural gas, coal, and oil [1]. To cope with the reduction in Russian gas and oil supplies, and the associated increase in prices, European regulations came into force, requiring member countries to achieve gas consumption savings of 15%, thus adopting countermeasures to prevent the occurrence of power shortages. This is pushing many states to compensate for the missing volumes of methane by reverting to the use of coal, despite the European Commission’s emphasis on transitioning to green energy sources. Concurrently, national governments are targeting societal and industrial sectors, such as residential areas, urban centers, and factories, to implement energy-saving measures.

In this regard, street lighting emerges as a particularly promising area for intervention, given its substantial impact on energy consumption. Street lighting alone can account for up to 40% of municipal electricity bills, contributing to 15% of global power consumption and 5% of greenhouse gas emissions [2,3]. This significance is further underscored by projections indicating a 50% increase in electricity usage for lighting purposes over the next two decades [2].

Indeed, the development and availability of heterogeneous sensing and communication technologies, jointly with the adoption of solid state light-emitting diode (LED) lamps, can reduce annual electricity demand for lighting by 40 to 60%, as shown by large-scale implementations [4,5,6]. As a matter of fact, simply replacing traditional 130W high-pressure sodium (HPS) lamps with 70W LED lamps, which are equivalent in terms of light output, results in savings of approximately 50%. This estimate, which is also confirmed by [7], is even conservative, as several papers claim that larger reductions in energy costs can be achieved [8,9]. In particular, Valentová et al. published a report on 106 test-beds from 17 European countries, showing an average energy saving of 59% compared with those of original installations [6].

However, there is still considerable potential for further improvements. Significant additional savings can be realized by integrating environmental sensing and remote control through a communication network into lighting infrastructures, enabling intelligent data processing to dynamically adjust lighting based on actual needs. For example, this could involve reducing lumen output (dimming) during periods of low traffic and good visibility conditions. The fundamental references in this regard are the EN 13201 standards for road lighting [10,11,12,13,14], which recognize that “*an appropriate lighting level at all times can only be ensured with adequate dimming control during off-peak hours*”, and the “GPP Revision of the EU Green Public Procurement Criteria for Road Lighting and traffic signals” [15], issued by the European Commission, which states that “*due to the multiple benefits of dimming, dimming controls must be installed in all cases unless, in exceptional circumstances, it can be demonstrated that the total cost of ownership would increase by installing dimming controls*”.

In Italy, the first document of the EN 13201 series, namely EN 13201-1, has been implemented and, above all, extended by the national standard UNI 11248 [16], which first introduces the possibility to dynamically adjust the light intensity by offering two alternative operating modes: (a) TAI, which dims the light based solely on real-time measurements of the traffic flow; and (b) FAI, which, in addition to traffic measurements, also requires the evaluation of meteorological conditions and road surface luminance.

In this paper, we will discuss the deployment of large-scale lighting infrastructures in Vietnam and Italy, which currently comprise hundreds/thousands streetlights integrating FAI, and we will report the measured energy savings that have actually been achieved. It is worth emphasizing that, in this manuscript, we do not focus on laboratory experiments or limited testbeds detached from real deployments. On the contrary, we will present measurements from several fully operational installations, each with hundreds/thousands of remotely controlled streetlights in real-world settings, that adhere to the latest regulations for public lighting and incorporate cutting-edge technologies. This has allowed us to evaluate their performance compared to more traditional lighting-control approaches. We will provide the results of measurements collected over a period of up to one year, demonstrating that significant energy savings can be achieved by equipping the lighting infrastructure with FAI capability, even up to 50% compared to conventional LED infrastructure.

However, energy saving is not the only factor to consider when it comes to lighting infrastructures. Since each smart streetlight must also be equipped with bidirectional communication technology, whose primary purpose is to enable communication with the infrastructure Control Center, it follows that a ubiquitous communication network originates from the lighting infrastructure itself, reaching every location where a smart streetlight is installed. Hence, in addition to enabling the remote control of each luminaire independently of the others, as well as the remote monitoring of its functional parameters (energy consumption, lamp temperature, electrical parameters), this ubiquitous network might also be used to collect data (concerning, for instance, vehicular traffic, air pollution, water flooding, etc.) gathered by sensors suitably mounted on the luminaires or in their proximity.

For example, smart streetlights, outfitted with sensors and wireless communication technologies, can detect nearby available parking spaces and notify interested motorists; they can notify garbage collectors when the neighborhood bins need to be emptied, or they can send valuable data on traffic flows to an urban traffic monitoring platform. They can also provide spatially dense measurements to infer the distribution of physical quantities such as air pollution, temperature, humidity, acoustic noise, and light intensity [17].

Ultimately, by embracing the smart-lighting paradigm, streetlights evolve from isolated elements into nodes of a pervasive, multifunctional citywide communication network capable of collecting data through sensors, transmitting information, and servicing IoT devices. The smart street lighting infrastructure emerges therefore as a dynamic platform, serving as the backbone for smart-city initiatives. This transformation allows cities to redefine the role of public lighting, offering new opportunities for service provision and revenue generation, rather than solely being perceived as a cost burden.

Moreover, it is worthwhile to remark that smart-lighting infrastructures can play a crucial role in supporting 5G (and beyond) cellular networks. The high data rates promised by 5G necessitate smaller cells and closer base stations, with an expected density of 40–50 base stations/km^2^ [18]. Integrating smart-lighting infrastructures with 5G networks presents a significant opportunity, as the density of streetlights in urban areas aligns well with the ultra-dense distribution requirements of current and future cellular networks. Moreover, hosting 5G base stations on streetlights would address site acquisition challenges and streamline their installation, as the power supply is readily available at each fixture.

The paper is organized as follows. Section 2 provides an overview of the existing literature on smart street lighting, while Section 3 outlines the general architecture and tasks associated with a smart-lighting infrastructure. In Section 4, we delve into the TAI and FAI strategies for dynamic light dimming, while Section 5 addresses some hidden issues within traditional lighting infrastructures that are identified and resolved through TAI/FAI. Section 6 introduces conventional light-control strategies, whose performances are compared with that of FAI. The real-world infrastructures discussed in this paper are introduced in Section 7, followed by numerical results in Section 8. Finally, conclusions are drawn in Section 9.

**Terminology**: In this paper, we will use the terms “light fixture”, “luminaire”, and “streetlight” interchangeably.

## 2. Related Works and Original Contributions

The dynamic control of light intensity and remote monitoring of streetlight operations have been extensively studied over the past years. Beginning with seminal papers on this topic [19,20,21,22,23,24], various remote-control strategies have been proposed, relying on communication between lighting fixtures and a Control Center, which serves as the hub for managing the lighting infrastructure. An overview of early research on smart street lighting systems is provided in [7].

The idea of adjusting light intensity based on actual context conditions has been explored since the pioneering era of smart lighting. For example, a dimming strategy based on the detection of moving objects was proposed in 2013 [25]. In the same year, the authors of [26] proposed a strategy for streetlight management based on environmental sensors and wireless communication technologies. Another research on smart street lighting based on vehicle detection appeared in 2014 [27], stating that energy savings could exceed 50% on low-traffic roads.

In the same period, the smart-city paradigm has gained prominence, and smart-lighting infrastructures have been increasingly considered pillars of this concept [28,29,30,31]. Specifically, in the smart-city framework, lighting infrastructures are meant to provide additional services, such as traffic flow forecasting and traffic-light regulation [32]. Smart-lighting infrastructures thus become citywide wireless sensor networks (WSNs), as in [33], where a WSNs-based solution to convert a conventional public lighting infrastructure into a smart one is discussed. Later, when the WSN concept faded away to be replaced by the broader Internet of Things (IoT) concept [34], lighting infrastructures were included in this new framework, as in [35,36,37,38,39,40,41,42]. This evolution has also raised security concerns, as public lighting is a critical asset that could be vulnerable to malicious attacks [43,44,45]. In this context, advancements in machine learning (ML) and deep learning (DL) techniques have provided methods to enhance the security of IoT systems [46,47,48]. Increased protection can also be achieved by adopting physical layer security techniques [49].

In recent years, smart urban lighting has gained considerable attention, leading to research on various aspects of this technology. Some studies focus on hardware design, including lamp drivers and light controllers [50,51,52,53,54], while others explore automatic, context-aware lighting control [35,55,56]. There are also investigations into energy efficiency [57,58,59,60,61,62], as well as studies addressing software and cloud-computing issues in smart-city scenarios [63,64,65]. Additionally, the research presents smart-lighting solutions utilizing specific communication technologies [66,67,68,69,70]. Regarding the possibility of adjusting luminous flux during the night to reduce it when low traffic is expected, an interesting paper demonstrates that significant savings can be achieved by selecting light intensity, also considering that a driver’s eye adapts to the vision in mesopic conditions [71].

When considering citywide testbeds, one of the largest examples is Smart Santander [72], where approximately 3000 IEEE 802.15.4 devices were deployed throughout the city of Santander, Spain, which makes the size of such a testbed comparable to the ones discussed in our manuscript. However, unlike our paper, [72] does not focus on smart lighting, so it does not offer specific insights into this application. Indeed, existing literature on public-lighting infrastructures often presents simulation results, such as [73,74,75,76], or reports findings from limited testbeds with few nodes [8,28,36,58,77], and occasionally from campus-wide installations [57,78,79].

To the best of the authors’ knowledge, the only paper discussing possible architectures, technologies, and performance of large-scale, smart-lighting infrastructures is our previous article [5]. The current work is based on our experience as researchers and entrepreneurs involved in designing and deploying smart street lighting infrastructures across Europe, Africa, the Gulf area, and Southeast Asia, with pilot installations commencing in 2014. Following the latest technical and regulatory developments in the field, in this article we focus on real lighting infrastructures capable of adapting the light intensity to traffic and environmental conditions in accordance with the EN 13201 [10,11,12,13,14] and UNI 11248 [16] standards, which have been installed in several countries in the past two years. This paper, in particular, will provide the following original contributions:

- a thorough discussion of the benefits of smart-lighting systems, some of which are truly unexpected as they only become evident when a traditional infrastructure is converted to a smart one, revealing subtle problems that in most cases were unknown to the infrastructure managers;
- the description of the hierarchical communication network actually implemented to make streetlights communicate with each other and with the Control Center. Both the architecture and the communication technologies will be discussed;
- an assessment of the energy consumption in actual large-scale lighting infrastructures in Vietnam and Italy, which adjust light intensity based on real-time luminance and traffic conditions using the FAI algorithm. It is emphasized that the measurements were collected over extended periods of up to one year and are compared to results obtained when a non-adaptive strategy is used;
- a comparison between the energy consumption of actual FAI-enabled lighting infrastructures in Vietnam and Italy and that of other currently adopted lighting-control techniques, including both static and dynamic methods.

## 3. Smart Public Lighting: Infrastructure Architecture and Main Tasks

**Figure 1:**
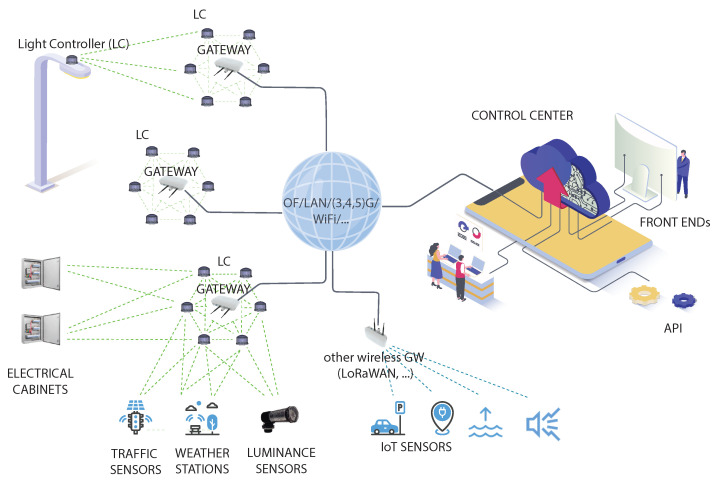
Architecture of a smart-lighting infrastructure.

Before delving into the specifics of the algorithms for dynamically adjusting light intensity and demonstrating the resulting cost savings, it is essential to first introduce the architecture of the smart-lighting infrastructure. Its main components, namely the Control Center, the communication network, the sensing devices, and the Light Controller (LC), are shown in Figure 1.

**Figure 2:**
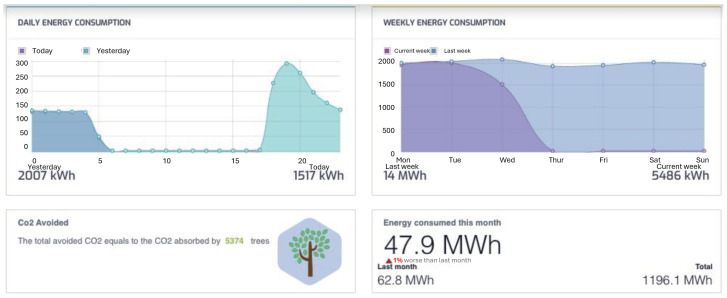
Example of a control panel for energy consumption monitoring.

**Control Center**: This serves as the headquarter for the infrastructure, where streetlights are managed and monitored for maintenance purposes. Here, the relevant data acquired by sensors placed on the light fixtures is collected to detect or predict faults and generate reports and statistics. It is also where the management of the entire infrastructure takes place, including the remote configuration of each light fixture independently of the others. For instance, Figure 2 illustrates the control panel of an application that tracks real-time energy consumption across the entire infrastructure. Similar interfaces are available for remotely monitoring the status and operation of each streetlight.

It is worth emphasizing that the Control Center should not be viewed as a physical location where staff personnel must go for management operations. Instead, the applications for controlling and monitoring the lighting infrastructure are run on servers, potentially third-party owned, connected not only to the lighting infrastructure itself but also to the Internet. This setup allows operations at the Control Center to be carried out remotely. For instance, the control panel shown in Figure 2 refers to the Control Center of the city of Tan An in Vietnam. Importantly, the cloud provider hosting the management and monitoring applications for this infrastructure is also located in Vietnam, for security reasons. This is a crucial aspect that must be carefully considered in the design of such critical infrastructures, as it directly impacts the safety of citizens.

It is worth emphasizing that the Control Center, being primarily composed of software applications, is constantly evolving to implement increasingly sophisticated control and management strategies [80]. For example, increasingly accurate predictive-maintenance techniques can be implemented over time, based on artificial intelligence algorithms that learn from the data collected by the infrastructure itself.

As anticipated, the exchange of information and commands between each streetlight and the Control Center takes place through the communication network, which must ensure adequate coverage of the entire area where streetlights are installed.

**Communication Network**: A key issue when designing an intelligent lighting infrastructure is how to establish a communication link between each streetlight (that is, each LC) and the Control Center. In principle, due to the fixed layout of lighting infrastructures, both wired and wireless technologies can be adopted. The reader is referred to [5] for an in-depth discussion of the pros and cons of each solution, as well as of the most suitable network architectures and communication technologies.

**Figure 3:**
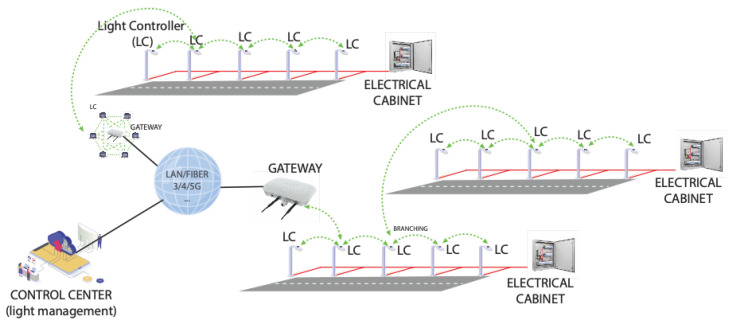
Bluetooth/IEEE802.15.4 mesh-based smart-lighting architecture.

**Figure 4:**
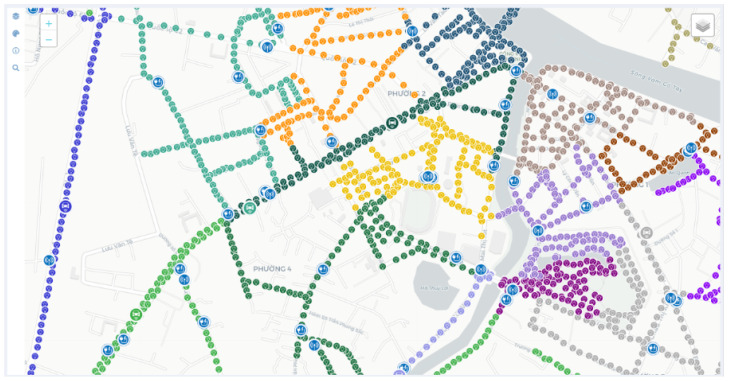
Tan An (Vietnam): example of network topology. Dots having the same color represent the locations of actual streetlights that belong to the same cluster.

Based on our direct field experience as designers and installers of street lighting infrastructures, we advocate for a two-tier hierarchical mesh as the optimal choice in most cases. According to this architecture, depicted in Figure 3, light fixtures that are geographically close (e.g., located on the same or neighboring streets) are grouped into clusters, wherein direct or hop-by-hop communications between lamps are enabled by a wireless mesh network implemented with short-range communication technologies, such as IEEE 802.15.4 [81] and Bluetooth. As for cluster formation, it is handled by the infrastructure designer, primarily based on the proximity of the streetlights. As an example, Figure 4 shows a map of a portion of the city of Tan An in Vietnam, where we have deployed an FAI-enabled smart-lighting infrastructure. Colored dots indicate the locations of actual streetlights, with dots of the same color representing streetlights that belong to the same cluster.

To enable communication with the Control Center, each cluster also incorporates a dedicated network device that, in addition to communicating with the streetlights in its cluster (either directly or hop-by-hop), is also equipped with a long-range communication technology (such as fibre optic, cellular, etc.). Serving as a gateway, this specialized device enables bidirectional information exchange and command transmission between the Control Center and all light fixtures within the cluster.

From an architectural standpoint, the short-range mesh network within each cluster represents the lower level of the hierarchical communication network, while the long-range network between the gateways and the Control Center constitutes the upper level. Importantly, this architecture does not suffer from coverage issues because each streetlight in a cluster is typically situated in close proximity to another, ensuring line-of-sight propagation conditions in most cases. As for the gateways, they are either directly connected to a wired network or strategically positioned to ensure robust connectivity if a wireless network (such as cellular) is used to reach the Control Center.

Finally, it is important to note that communications within a cluster do not require a subscription or payment to any network provider because the owner of the public lighting infrastructure also owns the short-range communication network, which is actually a private network. However, if a third-party network (e.g., cellular) is used to connect the gateways to the Control Center, a fee must be paid. Nonetheless, this is not a significant concern due to the low number of gateways required, typically one gateway for every 100 to 150 light fixtures.

The details of the network architecture and communication technologies used in the lighting infrastructures considered in this article will be provided in Section 7.

**Light Controllers**: These are the endpoints of the communication network, serving as counterparts to the Control Center, and are the intelligent components of streetlights. They execute the commands received from the Control Center and return locally sensed data. In addition, some LCs are equipped with traffic sensors (for TAI and FAI) as well as luminance and weather sensors (for FAI) that provide the necessary context information to dynamically change the light intensity. From a smart-city perspective, LCs might also be equipped with other types of sensor (for example, for monitor air quality), with their measurements transmitted to application-specific management centers (as depicted in Figure 1 under the label *IoT SENSORS*).

In summary, in a smart-lighting infrastructure, LCs are responsible for four main tasks:

- Task 1: to monitor the status of the lamp in real-time;
- Task 2: to monitor the electrical parameters of the fixture in real-time;
- Task 3: to transmit maintenance-relevant data to the Control Center and to receive and execute commands;
- Task 4: to drive the ballast, which is the electrical device used in lighting systems to regulate the electrical current flowing through the lamp, so as to dim the intensity of the light according to the sensed data.

Furthermore, a few LCs are assigned an additional task:

- Task 5: to collect and process the data gathered by traffic (for TAI and FAI) and luminance/weather (for FAI) sensors.

The measurements gathered in Task 5 are assumed to be representative of an entire area, referred to as TAI Area or FAI Area, depending on the availability of traffic measurements only (TAI) or also luminance and weather measurements (FAI), and are used to determine the dimming level of the streetlights within that area. Specifically, each streetlight belongs to one (and only one) TAI/FAI Area and adjusts its light intensity according to the measurements taken within that TAI/FAI Area.

Tasks 1, 2, and 3 enable timely maintenance, which results in shorter lighting outages and lower operating costs. Tasks 4 and 5 are, instead, closely related to the context-aware dynamic dimming, which is discussed in Section 4.

It is worth underlining that, collectively, the devices installed in an LC to enable Task 1 to Task 4 result in a power consumption of less than 1 W. Comparing it with the lamp’s power consumption, which typically ranges from 20 W (on lightly trafficked peripheral roads) to 150 W (on heavily trafficked roads), it results that the additional consumption required to equip streetlights with the TAI or FAI technology is at most a few percent.

## 4. Traffic Adaptive Installation (TAI) and Full Adaptive Installation (FAI)

In a smart infrastructure, the primary role of LCs is to adjust light levels based on specific criteria to achieve energy savings while ensuring driver safety. Initially, basic control systems were limited to turning lamps on at sunset and off at sunrise. However, these systems have evolved into highly advanced controllers capable of dynamically adjusting light intensity in real-time to suit the context. Nowadays, contemporary control systems can dynamically adjust lumen flux based on current vehicular traffic levels. In the most advanced versions, these systems consider real-time luminance measurements and weather information to account for actual visibility conditions. These sophisticated functions are developed in accordance with specific regulations, allowing for the dynamic reduction of light intensity under appropriate circumstances.

In particular, according to the European standard EN 13201-1 [10], each street designated for motorized traffic is assigned a specific lighting class based on various factors such as road significance, layout, expected vehicle speeds, traffic volume/composition, and environmental conditions. More precisely, each road is allocated one of six lighting classes, ranging from M1 to M6 in descending order of importance, each corresponding to a particular average luminance requirement. For instance, class M1 requires an average luminance of 2 cd/m^2^, while class M2 requires 1.5 cd/m^2^ and class M6 only requires 0.3 cd/m^2^.

In Italy, the EN 13201-1 standard has been implemented and enhanced through the UNI 11248 regulation, which introduces adaptive lighting strategies. This enables the design of systems capable of adjusting lighting conditions based on time-varying factors such as traffic flow and luminance, also taking into account weather conditions. Specifically, UNI 11248 introduces two different solutions for adaptive lighting management:

- Traffic Adaptive Installation (TAI), where only the traffic volume is measured;
- Full Adaptive Installation (FAI), where, in addition to traffic, the luminance of the road surface is also measured. This ensures that the required lighting level is always met. Weather conditions are also assessed to adopt specific lighting settings if risky conditions are detected.

Needless to say, both strategies require the adoption of sensors placed on appropriately chosen streetlights. These sensors provide the LC with raw data to assess the amount of traffic (for both TAI and FAI), as well as luminance and weather conditions (if FAI is adopted). The latter, in particular, are assessed by means of rain, snow, and fog sensors. Furthermore, the need to transmit such data in real-time to other streetlights in the same TAI/FAI Area must be met to ensure uniform and simultaneous dimming.

Hereafter, additional information on TAI and FAI is provided, with more emphasis on FAI, which can be considered an enhanced version of TAI.

### 4.1. Traffic Adaptive Installations (TAIs)

Lighting infrastructures that comply with TAI adjust the light intensity based on the estimated hourly traffic flow. This estimation, referred to as the *sample*, is derived by converting the number of vehicles travelling on the road during a 10 min period (counting period) into an hourly basis. This conversion is achieved by multiplying the count obtained during the counting period by 6. The estimation process is carried out continuously, resulting in a new *sample* being derived every 10 min.

The lighting class can be downgraded by one (e.g., from M2 to M3) if the estimated hourly traffic is lower than 50% of the (a priori defined) nominal value of the traffic flow for the considered road, whereas a downgrade of two lighting classes is possible when the estimated hourly traffic is lower than 75% of the nominal value. More precisely, dimming is allowed when two consecutive *samples* are below such thresholds, whereas the increase in lighting class is triggered as soon as a *sample* requiring the higher class is encountered.

### 4.2. Full Adaptive Installations (FAIs)

In contrast to TAI, which employs discrete stepwise adjustments, FAI updates the lumen output progressively and continuously between lighting classes to achieve maximum energy savings. In this case, the dimming level is determined by considering traffic conditions, road surface luminance, and weather conditions collectively. In addition, FAI allows dimming of up to three lighting classes for the busiest roads: one lighting class when the traffic is less than 50% of the nominal volume, two lighting classes when the traffic falls below 75% of the nominal volume, and three lighting classes when the traffic is less than 12.5%.

**Sampling of hourly traffic flow**. The hourly traffic flow is estimated through the following procedure:

(a) counting for 1 min (counting period) the number of passing vehicles;
(b) calculation of the traffic *sample* by converting the result of step (a) to an hourly basis (that is, by multiplying by 60);
(c) estimating the hourly traffic flow as the arithmetic mean of the last 10 *samples*;
(d) From now on, repeat the vehicle count every minute (point (a)), calculate the traffic *sample* (point (b)), and discard the oldest *sample* and add the last one to continue estimating the hourly traffic flow by averaging over ten *samples* (moving average).

**Decrease or normal increase in light intensity**. The estimated hourly traffic flow determines the required lumen output, which is obtained by linear interpolation between the nominal lighting class (corresponding to 100% of the hourly traffic flow) and the last allowed class.

When the hourly traffic flow exceeds 100% of the expected volume for the considered road, it is possible to downgrade by one lighting class from the nominal level. This decision stems from the observation that, in such situations, vehicle speeds tend to decrease due to high traffic density. Conversely, when the hourly traffic falls below the threshold corresponding to the lowest permitted lighting class for the road, the lighting class cannot be further reduced.

**Fast increase in light intensity**. If three consecutive *samples* are greater than 20% of the moving average calculated at point (d), the system must immediately adjust to the corresponding light intensity obtained through linear interpolation between the value dictated by the lighting category of the street (traffic flow equal to 100%) and the last identified operating category.

**Stabilization of road surface illumination in real-time**. Continuous adjustment of the luminous flux emitted by the luminaires is carried out to ensure that the luminance on the road surface, measured by dedicated sensors, always corresponds, within the limits of measurement uncertainty, to the design values. The variations in luminance that the system must compensate for are determined essentially by the following:

- decay of luminous flux generated by light sources due to ageing or dirt on optics;
- variation of reflection characteristics of the road surface;
- power supply fluctuations;
- change in weather conditions.

The measurement of road surface luminance for adaptive lighting systems is regulated by Appendix D of EN 13201-4 [13].

**Weather conditions**. The impact of adverse weather conditions (wet road, fog, snow) on the effectiveness of the lighting system must be evaluated by the infrastructure designer. This involves performing a risk analysis and deciding which control strategies to implement when these conditions arise (no change, reduction, or increase in the intensity of light).

### 4.3. Logical Network Architecture

As anticipated in Section 3, when TAI or FAI are implemented, streetlights are organized into distinct clusters, each controlled by a gateway. A cluster typically comprises 100 to 150 streetlights, with a typical distance between adjacent streetlights ranging from 25 to 30 m. The number of communication hops between the gateway and the outermost light fixtures of a cluster is typically between 5 and 10.

Streetlights covering streets with the same lighting class are grouped into a TAI or FAI Area, depending on whether the infrastructure is TAI-enabled or FAI-enabled. The number of these areas varies depending on the city’s size, ranging from just a few to a few tenths. Each TAI and FAI Area is equipped with a device hosting a traffic sensor. In FAI-enabled infrastructures, at least one luminance and weather sensor is also required.

Based on the sensed data (traffic only for TAI, traffic, luminance, and weather for FAI), all streetlights within the same TAI/FAI Area, possibly belonging to different clusters, adjust their light intensity accordingly. It is worth noting that clusters are logical entities and can correspond to various network topologies. For example, a cluster could include luminaries distributed along a straight road or covering a circular area, such as in a historic center and its internal roads. At a higher hierarchical level, the logical grouping of streetlights that share the same lighting class in a TAI or FAI Area reflects the segmentation of roads based on their categories and is completely independent of the physical arrangement of clusters. The specific organization into TAI/FAI Areas is determined by the infrastructure designer, who conducts a risk analysis and evaluates the size, extent, features, and dimming strategies according to the characteristics of the area.

**Remark**: According to FAI, the decision on the appropriate light intensity for a group of streetlights is based on measurements of traffic volume, luminance, and weather conditions. Clearly, these measurements (and the resulting decision on light intensity) must be taken near the interested streetlights to ensure that their light output is appropriate for the actual conditions in their proximity. Consequently, FAI inherently calls for edge computing, as data processing and decision-making occur close to the locations where these decisions take effect. Contrarily, resorting to cloud computing—involving the transmission of all local measurements to the Control Center for processing and decision-making—would increase the load on the long-range communication network without offering any additional benefit.

## 5. Solving Unexpected Issues through FAI-Enabled Lighting Systems

Upgrading a traditional lighting infrastructure to enable FAI often reveals underlying issues within the pre-existing infrastructure that were previously unknown. Regrettably, these problems commonly lead to substantial energy over-consumption and a subsequent waste of money. Below, we will delve into the most critical issues and explain how they can be easily resolved by implementing an FAI-enabled lighting infrastructure.

### 5.1. Energy Over-Consumption Due to Over-Performing Lamps

Lamps used in public luminaires are expected to provide a certain luminous flux with a predetermined energy consumption. However, when upgrading existing infrastructures to enable FAI, it is often discovered that some lamps have generated more light than required by regulations, resulting in unwanted energy over-consumption.

This over-consumption is usually due to a mismatch between the characteristics of the lamps provided by the supplier and those specified by the municipality during the bidding process. Indeed, it sometimes happens that, at the time of supply, which can occur significantly later than the initial tender documents, the lamps provided by the supplier are more efficient than those initially agreed upon, owing to technological evolution. This means that when powered according to the initial design, they output more light than required, resulting in over-consumption.

Streetlights with energy metering and communication capabilities, such as those that make up the smart-lighting infrastructure discussed here, make it possible to reveal and solve this issue by taking action on each lamp. Firstly, all lamps within the same stock that exhibit over-consumption are detected based on the similarity of their anomalous dimming/consumption curves, which can be easily derived for each lamp by the Control Center. Then, once the actual photometric curve of the anomalous lamps has been determined through measurements, deviations from the ideal curve can be compensated for by adjusting the dimming profile adopted by the LC. This approach allows for significant energy savings across the entire installation.

It may also happen that lamps with higher energy consumption and consequently higher luminous flux than required are installed on streetlights. This might be due to errors or because the appropriate lamp is not in stock. For instance, during installation activities, we encountered lamps expected to consume 53 W actually consuming 57.4 W. In a TAI/FAI-enabled infrastructure, this issue, usually unknown to infrastructure managers, can be easily spotted thanks to power consumption measurements that are regularly transmitted to the Control Center, which can then remotely adjust the power supply of the lamp accordingly.

### 5.2. Energy Over-Consumption Due to Non-Linear Dimming

In each streetlight, the device responsible for adjusting the dimming level is the LC, which sends a control signal to the lamp ballast for this purpose via a wired link. In this regard, there are two traditional ways to establish a communication link between the LC and the ballast: by means of an analog signal or through a digital bus. In the former case, the LC outputs a control signal in the range [0–10 V], whose voltage is interpreted by the ballast as the percentage of light to be supplied to the lamp (e.g., 1 V = 10%, 5 V = 50%, 10 V = 100%) [82]. This analog controller, which is compliant with the American National Standard for Lighting Systems [82], is usually referred to as *NEMA* (founded in 1926, the National Electrical Manufacturer’s Association (NEMA) is an ANSI-accredited Standards Developing Organization made up of business leaders, electrical experts, engineers, scientists, and technicians [83]). When digital control is adopted, instead, the most common choices are D4i or DALI2-based drivers [84,85].

As of today, the international market is divided into two segments: The European market has almost completely shifted towards the use of D4i or DALI2, whereas in Southeast Asia, as well as markets under the influence of British regulations, still widely use 0–10 V NEMA controllers.

**Figure 5:**
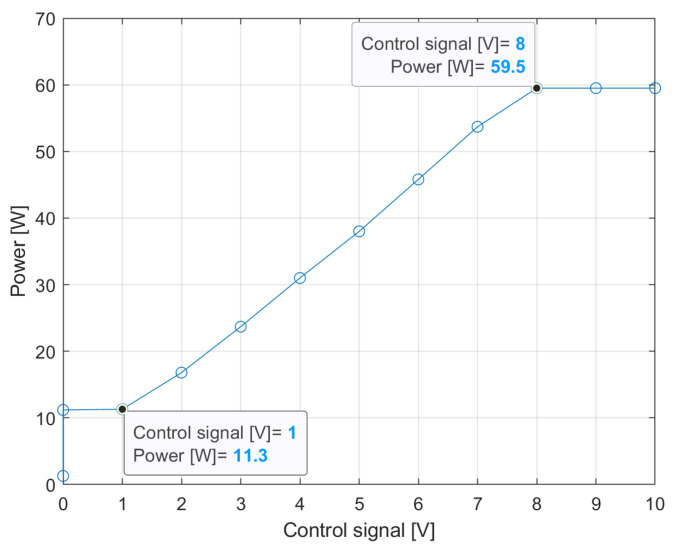
Measured power consumption of a 52 W lamp as a function of the control signal used to set the dimming level.

Obviously, the prerequisite for the NEMA controller to operate properly is that the response of the ballast to the [0–10 V] control signal is linear in the same interval, so that, for example, 90% dimming is applied when the control signal is 9 V. Unfortunately, LED drivers can sometimes have a standard setting to dim following a control signal in the range of [1–10 V], [0–9 V], [1–9 V], or [1–8 V]. To exemplify, Figure 5 shows the measured power consumption as a function of the control signal for a 52 W lamp used in a lighting infrastructure we were enabling with FAI. Even though the manufacturer should have provided a driver with a [0–10 V] pilot, the non-linear characteristic is clearly evident, as there is no reduction in luminous intensity until the control signal drops below 8 V, despite this voltage level being expected to correspond to a 20% dimming.

Of course, this discrepancy between what is dictated by the LC and the actual response of the lamp causes energy over-consumption, which is generally not known to infrastructure managers. Also in this case, the evolution towards a smart infrastructure with power metering and communication capabilities allows the problem to be identified immediately, enabling the calibration of the LC output for a linear response.

Incidentally, Figure 5 also shows that, in the absence of dimming (control signal = 10 V, corresponding to full lighting), the power drawn by the lamp (59.5 W) is higher than expected (52 W). This is the over-consumption phenomenon discussed in the previous section.

## 6. Reference Dimming Strategies for Evaluating Energy Saving

In the numerical results section, we will show the energy savings achievable with FAI-enabled lighting infrastructures currently operating in Italy and Vietnam. To this end, in the following we will briefly introduce the strategies universally adopted for managing streetlight intensity, namely “astronomical clock”, ”virtual midnight”, and ”motion-sensing”, which will serve as references for comparison with FAI.

**Astronomical clock**: Astronomical clocks are used to regulate streetlights by synchronizing their operation with the natural cycles of daylight and darkness. These clocks are specifically programmed to synchronize streetlight operation timings based on predefined tables, which specify the sunrise and sunset times in the region where they are installed. Notably, they account for seasonal variations in daylight length, ensuring that streetlights adapt accordingly throughout the year. However, during the “on” period, the light intensity remains constant, set at the maximum allowed by the lamp.

**Virtual midnight**: In order to achieve energy savings, a luminaire can be equipped with a programmable driver that dims the light following a specific time profile.

According to the “virtual midnight” strategy, the luminaire adjusts its luminous flux through a self-learning process that, based on switch-on and switch-off times from previous days, determines the hypothetical ”virtual midnight”. This is the central instant within the period from switch-on (at sunset) to switch-off (at sunrise) of the luminaires. In turn, the switch-on and switch-off times are determined by astronomical clocks (or even twilight switches) installed in the electrical cabinet providing power to the luminaires.

**Figure 6:**
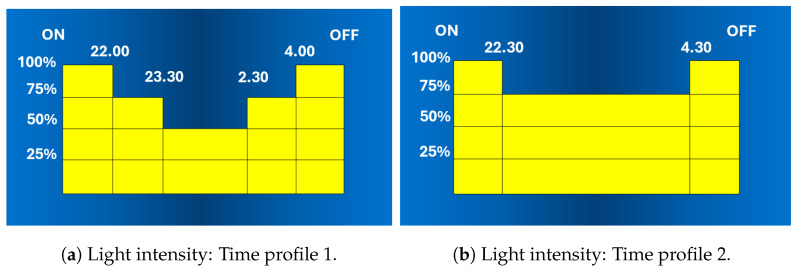
Virtual midnight: Examples of light-time profiles.

The “virtual midnight” serves as the reference point for adjusting the light according to the desired profile. For instance, a 50% reduction in light intensity might be applied three hours before the “virtual midnight”, followed by a return to full illumination four hours after. Typically, these settings can be adjusted to meet specific needs and create custom profiles. In this regard, Figure 6 shows two examples of light dimming variations over time, each corresponding to a custom profile.

Although the “virtual midnight” strategy is an improvement over simple on/off switching dictated solely by the astronomical clock, it is a static approach and therefore unable to adapt the light intensity to actual traffic and visibility conditions. This could potentially compromise road safety by reducing the light intensity during high-traffic events.

**Motion-sensing**: This strategy relies on motion sensors embedded in each streetlight. When a sensor detects motion nearby, the light intensity emitted by the lamp is increased to the maximum expected value. Additionally, this information is transmitted through a communication network to neighboring streetlights, causing them to turn on as well. This motion-driven sensing is often known as the cascading effect. When motion is no longer detected by any sensor, a timer is triggered, and once it expires, the lighting is adjusted to the lower level expected for that road.

## 7. The Lighting Infrastructures Considered for Performance Assessment

The measurements that will be presented in Section 8 refer to three lighting infrastructures enabled with FAI that have been installed in Vietnam and Italy. These findings are representative of outcomes observed in other infrastructures where we have deployed tens of thousands of luminaires and are therefore broadly applicable. Additional details are provided below.

### 7.1. Real-World Installations under Investigation

**Scenario 1. Tan An (Vietnam)**: This scenario consists of a city with 3815 FAI-enabled luminaires. The reported measurements pertain to a subset of 878 luminaires installed in “phase-one”, for which we extensively collected data. We would like to underline that, to date, after completing “phase-two”, which brought the total to 3815 FAI-enabled lamps, the overall energy savings are consistent with those observed during “phase-one”.

The 878 FAI-enabled luminaires are divided into 8 clusters and 4 FAI Areas, covering approximately 22 km of roads. The measurements presented in Section 8 for this scenario are based on measurements taken over a period of 6 months.

**Scenario 2. Meda (Italy)**: This infrastructure consists of 2957 FAI-enabled luminaires, divided into 31 clusters and 7 FAI Areas, covering approximately 72 km of roads. The numerical findings showcased in Section 8 are derived from data collected over a timeframe of 3 months.

**Scenario 3. Cesena (Italy)**: This is a pilot installation comprising 32 FAI-enabled luminaires within one cluster and one FAI Area, covering a 0.8 km stretch of road. While less complex than previous scenarios, it was considered due to the availability of measurements collected over a 16-month period in a seasonal setting. These measurements allow for accounting for the significant difference in daylight duration between winter and summer, a factor that does not apply to Scenario 1 due to Tan An (Vietnam) being much closer to the equator compared to Cesena (Italy).

### 7.2. The Adopted Communication Technologies

As mentioned in Section 3, the communication network for interconnecting individual light fixtures to the Control Center is a two-level hierarchical type: the lower layer, realized through short-range wireless communication technologies, consists of a mesh network within the clusters, whereas the upper layer handles the long-range communications between the clusters’ gateways and the Control Center. Regarding the FAI-enabled infrastructures discussed in this paper, the details on the technologies adopted in each layer are provided hereafter.

**Short-range network**. The mesh network within each cluster has been implemented using IEEE 802.15.4 wireless technology [81], which supports short-range, low-throughput applications in wireless personal area networks (WPANs)). The standard specifies the physical and medium access control (MAC) layers for low-rate wireless PANs (LR-WPANs) and is maintained by the IEEE 802.15 Working Group. It is the basis for the Zigbee, WirelessHART, ISA100.11a, 6LoWPAN, MiWi, Thread, and SNAP specifications, each of which further extends the standard by developing the higher layers, which are not defined by the IEEE 802.15.4 standard.

At the physical layer, different modulation schemes can be used, providing over-the-air speeds of up to 250 kbps in specific licence-free bands (depending on the geographical region) at 800, 900, and 2400 MHz. The radio coverage ranges from tens to hundreds of metres, depending on the propagation conditions and the maximum power transmission. In our solution, the LC dynamically adjusts the transmission power to reach a maximum level of 20 dBm EIRP, ensuring coverage distances greater than 1 km.

The MAC protocol, responsible for managing access to the channel, is based on the carrier sense multiple access with collision avoidance (CSMA/CA) strategy. One important operational aspect of the IEEE 802.15.4 technology is its ability to define different types of devices: Reduced Function Devices, which have limited capabilities and cannot forward data, and Full Function Devices, that can act as dynamic routers in a mesh topology. Different solutions may be adopted at the network layer to create mesh topologies (e.g., Zigbee, 6LoWPAN, etc.); in our implementations, we used a proprietary routing algorithm inspired by the many-to-one routing strategy defined in Zigbee [86]. Additionally, a fundamental aspect that cannot be ignored for a critical asset like a public lighting infrastructure is security. Our infrastructure includes a security co-processor that provides hardware-based 128-bit AES-CCM modes as specified by the IEEE 802.15.4-2006 standard, which adopts both encryption (ENC) and Message Integrity Code (MIC). Specifically, this includes encryption and authentication covered by the MIC–32/-64/-128, ENC, and ENC-MIC-32/-64/-128 modes of operation. All data transferred to and from the network is protected using this security co-processor.

According to IEEE 802.15.4 terminology, the gateway is called a Coordinator, and the cluster of devices controlled by a Coordinator is called a personal area network (PAN). In our deployments, to avoid mutual interference, adjacent PANs (i.e., adjacent clusters) work on different channels.

Within a cluster, information propagates from the Coordinator to the border streetlights (and vice versa) by jumping from one streetlight to another. Since streetlights are usually placed along roads, in most cases the resulting topology consists of linear segments and a few branches, as shown in Figure 3.

In actual installations, IEEE802.15.4 transceivers have a transmission range that covers not only the adjacent light fixtures but also a number of neighboring ones. This makes the system inherently robust to possible failures, as a given streetlight is usually reachable by more than one neighbor.

Regarding the amount of generated data traffic, packets exchanged within a cluster have variable-length payloads that depend on the type of information they carry. This may include light intensity instructions for streetlights, status reports sent by each streetlight to the Control Center for monitoring and maintenance purposes, event notifications from additional sensors (e.g., the presence of cyclists on a nearby cycling lane), fault notifications, and reception acknowledgments. The maximum payload length is 64 bytes.

The timing and volume of these transmissions depend on several factors, such as the number of streetlights in a cluster, the topology of the mesh network, the specific observation period (evening, late night, early morning), the particular streets covered by the cluster, and whether it is a weekday or not. Therefore, providing a meaningful assessment of the overall data traffic volume is challenging. Focusing on the Coordinator of each cluster, which manages communications to and from the cluster, it is expected to handle a few packets per second.

**Long-range network**. The long-range network aims to connect the clusters’ Coordinators to the Control Center. To this end, our installations rely on various media, including the cellular network—whether 3G, 4G, or 5G, depending on the available technology on-site, as well as fiber or LAN if available in the area.

It should also be noted that streetlights could use cellular technology to communicate directly with the Control Center, thereby eliminating the need for clusters, Coordinators, and short-range communication technology. However, this does not appear to be the most appropriate choice for TAI/FAI-enabled lighting infrastructures, both for economic and technological reasons.

From an economic standpoint, streetlights that continuously exchange real-time data cannot be compared to traditional IoT devices, which generate sporadic data traffic and benefit from low economic rates, typically just a few dollars per device per year. In contrast, the high cost imposed by cellular operators to support the extensive data traffic generated by the entire lighting infrastructure would render the project economically unsustainable, given that its primary objective is to reduce expenses. If a significant portion of the yearly budget for public lighting is spent on telecommunication fees, the project loses its purpose.

From a technical perspective, two key elements must be considered. The first is the lifespan of a lighting project, which should ensure technological continuity for 10–15 years. This timeframe cannot currently be guaranteed by telecom providers, who rapidly evolve their networks and render technologies obsolete within shorter intervals. The second element is connectivity, which is fundamental for intelligent lighting infrastructures. Streetlights not experiencing adequate connection quality cannot be managed or monitored effectively. This issue is particularly critical because, although cellular networks are widespread, providing reliable connectivity to all streetlights might be unfeasible in dense urban environments. Buildings or other obstacles can hinder signal propagation, thus compromising communication dependability in certain locations. Moreover, this situation can arise at any time with the construction of new buildings. Consequently, cellular technology, although generally available and easily accessible, and despite being used by some players for daily diagnostics, is not suitable for providing reliable connectivity to lighting devices within a cluster if the goal is to enable TAI or FAI.

Using the cellular network to provide long-range connectivity to Coordinators, on the other hand, is much less critical. In fact, Coordinators are far fewer in number than streetlights and can be conveniently placed in areas with adequate cellular coverage. Then, under the coverage umbrella of the cellular network, connecting a Coordinator to the Control Center is as simple as equipping it with a cellular radio interface and turning it on. It will immediately connect with the cellular network that is already in place.

## 8. Street Lighting: Numerical Results from Large-Scale, Long-Term Implementations

In this section we show the advantages, in terms of energy savings, that can be achieved by transforming a traditional lighting infrastructure into an FAI-enabled one. We will first focus on the energy consumption over one night, which allows one to visualize the savings generated by FAI on an hourly basis throughout the entire nighttime. Next, we will investigate energy consumption of citywide infrastructures over several months with daily resolution, comparing installations at different latitudes (Vietnam and Italy) that feature varying daylight duration during the observed period. Finally, we will compare the energy savings achieved by FAI with those of the alternative dimming strategies introduced in Section 6.

### Large-Scale, Long-Term Measurements and Comparisons

**Figure 7:**
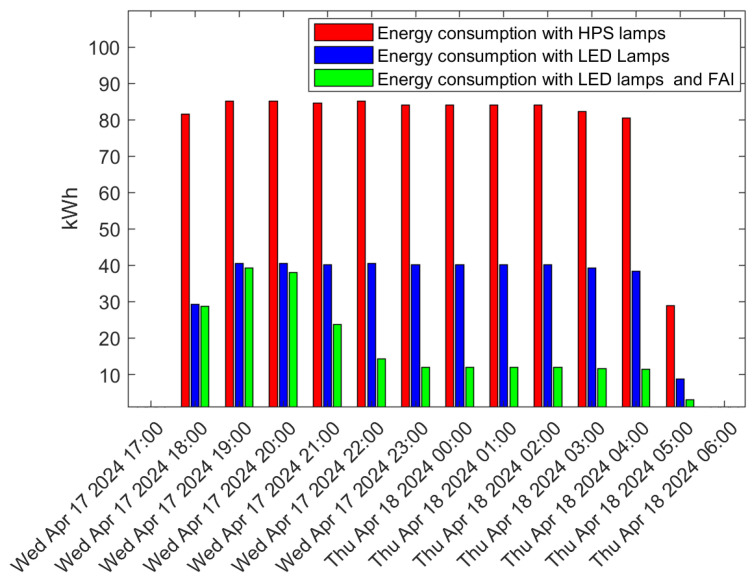
Energy consumption per hour with HPS lamps (no dimming), LED lamps (no dimming), and LED lamps with FAI. An FAI Area consisting of 300 streetlights in Tan An (Vietnam) was considered, covering equivalent M2 streets.

First of all, it must be emphasized that, for dimming to be possible, the lighting infrastructure must operate with LED lamps, as conventional HPS lamps are not dimmable. Already in itself, replacing HPS lamps with LED lamps, which can generate the same luminous flux with lower power consumption, results in significant energy savings, which can be further increased by enabling the infrastructure to FAI. This is clearly evident in Figure 7, which shows the energy drawn by a cluster of 300 streetlights in Tan An (Scenario 1) during an entire night when using 275 W (on average) HPS lamps or 135 W (on average) LED lamps with and without FAI.

It is noted that upgrading a conventional HPS infrastructure to an LED one results in a reduction in energy consumption by approximately 50%, decreasing from 85 kWh to about 40 kWh in each of the one-hour time intervals represented in the figure. Additional remarkable savings can be achieved with the implementation of FAI, particularly noticeable after 11 pm. By enabling FAI, energy consumption can decrease by up to 70% compared to non-dimmable LED lamps, resulting in a final consumption of 12 kWh per hour. This saving was achieved by reducing the lighting class by four levels when low traffic conditions are detected. We highlight that reducing the lighting class by four levels is permitted in Asian countries, where the installation under consideration is located.

**Figure 8:**
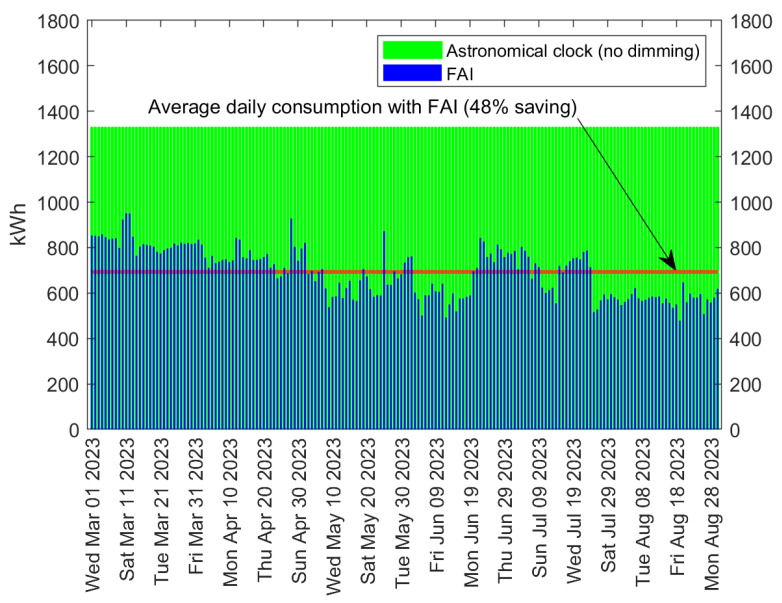
Daily energy consumption with LED lamps in Tan An (Vietnam), both without dimming and with FAI; 878 streetlights were considered, covering M2 streets.

Figure 8 refers again to the Tan An installation, which at the time of the reported measurements consisted of 878 FAI-enabled streetlights covering M2 streets. The figure shows the daily energy consumption over a period of six months, from 1 March 2023, to 31 August 2023. In this time interval, the variation in daylight duration in Tan An is approximately 20 min, resulting in the streetlights being “on” almost constantly for 12 h from March to August. Consequently, adopting the astronomical clock (that is, without adaptive dimming), the daily energy consumption remains constant at approximately 1300 kWh, as clearly seen in Figure 8.

Conversely, with the adoption of FAI, energy consumption exhibits day-by-day fluctuations, depending on vehicular traffic, luminance, and weather conditions, and, most importantly, a significant reduction. Specifically, during the observed period, energy savings of approximately 48% were achieved, with an average daily consumption of 693 kWh, as illustrated by the horizontal line in Figure 8. Indeed, Figure 8 allows an immediate qualitative assessment of the benefits introduced by FAI, as the light-green area represents the saved energy. It is worth underlining that, given the remarkable advantages provided by FAI, the Tan An municipality decided to progressively increase the amount of FAI-enabled streetlights, which in January 2024 was already 3815.

**Figure 9:**
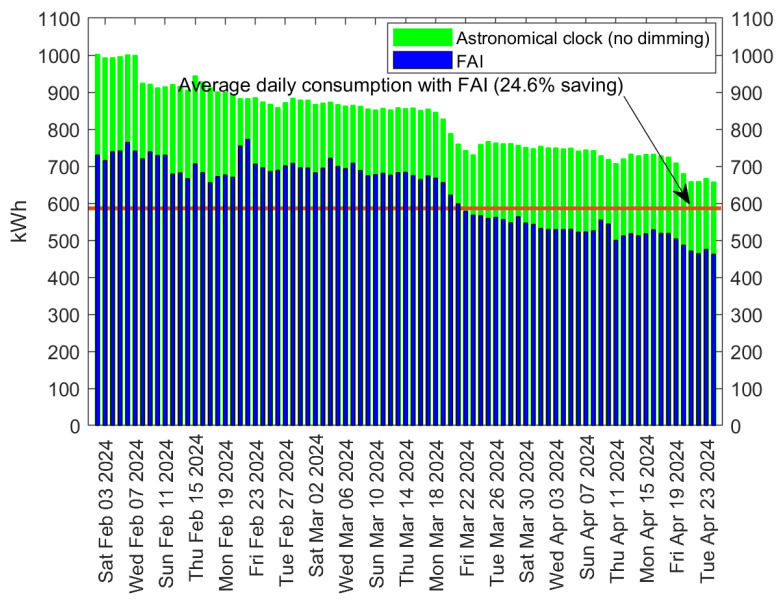
Daily energy consumption with LED lamps in Meda (Italy), both without dimming and with FAI; 2957 streetlights were considered, belonging to M4 and M5 classes (low-traffic streets).

Figure 9 refers to Scenario 2, specifically focusing on the city of Meda in Italy. The 2957 luminaries, equipped with 27 W (on average) LED lamps, are placed in streets classified as M4 and M5, which permit a downgrade of the lighting class by one level only. This means that the minimum light intensity is set at 75% of full lighting, which significantly limits the achievable savings. Similar to Figure 8, Figure 9 illustrates the daily energy consumption over time for both LED lamps with an astronomical clock and with FAI. In this case, measurements were taken over a period of roughly three months, from 3 February 2024, to 26 April 2024.

**Figure 10:**
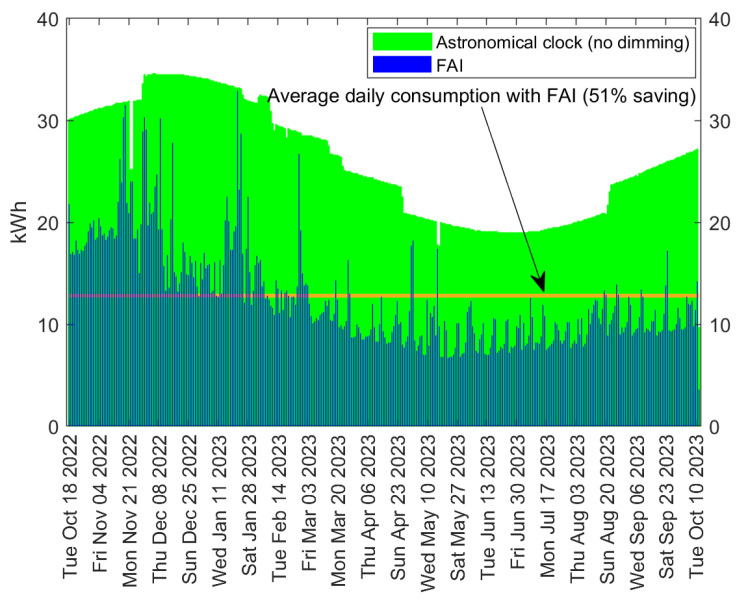
Daily energy consumption with LED lamps in Cesena (Italy), both without dimming and with FAI; 32 streetlights were considered that cover one M3 street.

In contrast to Figure 8, this scenario exhibits a decreasing trend in energy consumption over time, owing to the increase in daylight duration from February to April, which is noticeable in Italy. More importantly, the average daily consumption with the astronomical clock amounts to 778 kWh, which decreases to 586 kWh with the implementation of FAI, resulting in an overall saving of 24.6%. Remarkably, even with the restriction of only one downgrade in light class, the savings remain significant. To better account for variations in daylight that occur at latitudes farther from the equator compared to Tan An (Scenario 1), we extend our investigation considering a pilot installation in Cesena, Italy, where we collected measurements for a period of one year, thus encompassing all seasons. This scenario, namely Scenario 3, consists of a pilot infrastructure comprising 32 streetlights equipped with 76 W (on average) LED lamps belonging to one cluster and one FAI Area. The results are shown in Figure 10, which showcases the daily energy consumption from 12 October 2022, to 12 October 2023. Similar to Figure 8 and Figure 9, also in this case the daily energy consumption is reported for both LED lamps with astronomical clocks and with FAI.

It is worth noticing that, in Cesena, the variation in daylight duration over the year is approximately 6 h and 40 min, which is reflected in the fluctuating trend of energy consumption shown in the figure, with the maximum and minimum occurring in December and June, respectively. Over the entire period, the average daily consumption with FAI activated amounts to 12.8 kWh, resulting in a 51% reduction compared to the average consumption with the astronomical clock, which stands at 26 kW.

As anticipated in Section 6, other strategies have been devised to save energy by reducing the light intensity of streetlights during the night, namely the virtual midnight and motion-sensing strategies. Below, we compare the energy consumption achieved by these strategies when adopted in three actual streets belonging to different light classes, with that achieved by FAI. Also in this case, the benchmark for our comparison is the light control based on the astronomical clock, which, being a static strategy, merely turns on the streetlights at dusk and off at dawn.

Specifically, in our investigation based on actual in-field measurements, we considered as a reference a single streetlight with a 100 W lamp, whose light intensity is controlled either by FAI, virtual midnight, motion-sensing, or by means of an astronomical clock. Since FAI and motion-sensing adjust light intensity based on street traffic, the performance of these two strategies depends on the actual vehicular flow. For this reason, we monitored the movements on three streets, classified as M3, M4, and M5, over a period of several months in two different cities where FAI-enabled streetlights were installed: Cesena (Italy) for M3 and Biassono (Italy) for M4 and M5. These traffic traces are used to assess the energy consumption in the case where the motion sensing strategy is used. The results for the comparison were obtained and are reported in Table 1.

- **FAI**. The energy consumption of the FAI-enabled reference streetlight for the entire observation period was measured on each of the three monitored streets. The measurement results are reported in the first column of Table 1. The table also indicates the duration of the monitoring period. Since FAI is the only strategy sensitive to rain, we also computed what the consumption would have been without the rain sensor, which is reported in the second column of the table. Clearly, since in the case of rain FAI increases the light intensity regardless of the amount of traffic, the values in the second column are lower compared to those in the first.
- **Virtual midnight**. For the same streets and during the same periods, we also computed the energy consumption in the case where the virtual midnight strategy was adopted. The results are reported in the third column of Table 1. Specifically, for the monitored M3 and M4 streets, the time profile shown in Figure 6a was adopted, whereas for the M5 street, the adopted profile was that shown in Figure 6b. The comparison with FAI reveals the worse performance of the virtual midnight strategy, especially evident on the M3 and M4 streets, where FAI is able to almost halve the consumption. On the other hand, the effectiveness of FAI on the M5 street, although evident, is less significant. This was expected because, in M5 streets, FAI can dim the light by up to 75% of the maximum value, aligning with the time profile adopted by virtual midnight, as shown in Figure 6b.
- **Motion-sensing**. The traffic measured on the three roads in Cesena (class M3) and Biassono (class M4 and M5) was the input to calculate the energy consumption of the reference streetlight, which was assumed to provide full light as soon as a vehicle is detected and, when no traffic is sensed, to reduce light intensity to 30% of the maximum on the M3 and M4 streets, and to 75% of the maximum for the M5 street. According to this strategy, when the motion sensor no longer detects vehicles, a timer is activated, and the light dims when the timer expires, assuming no other vehicles are detected. In this regard, Table 1 provides the energy consumption assuming four possible durations of the timer, namely, 1 min, 2 min, 3 min, and 5 min. It is observed that, regardless of the timer duration, the motion-sensing strategy is far from achieving the energy savings guaranteed by FAI on the M3 and M4 streets, being worse, albeit to a lesser extent, also in the M5 street. Remarkably, the motion-sensing strategy appears to be less effective than the virtual midnight strategy in the majority of cases.
- **Astronomical clock**. As is known, the astronomical clock does not perform any light adjustment. Therefore, not surprisingly, the data reported in the last column of Table 1 show that it is the worst solution in terms of energy consumption.

In conclusion, Table 2 provides a comprehensive overview of the percentage of energy savings of FAI, virtual-midnight, and motion-sensing relative to the astronomical clock. The data demonstrate that FAI stands out as the most efficient solution among all the considered strategies. The energy savings offered by FAI surpass those achieved with both the virtual midnight and motion-sensing strategies by a significant margin, exceeding twice the savings realized by these alternatives. This reaffirms FAI’s effectiveness in optimizing energy usage and underscores its potential as a superior choice for modern lighting infrastructure, due to its joint consideration of traffic, road surface luminance, weather, and continuous dimming.

**Commentary.** The energy savings achieved with FAI depend on several factors, one of which is nighttime duration. The results presented in this paper refer to installations in Vietnam and Italy. The former, being close to the equator, experiences a nearly constant nighttime duration throughout the year, while Italy shows significant differences between summer and winter. However, investigations at other latitudes could provide additional insights into the benefits that FAI can offer depending on the installation site. This investigation is left for future work.

## 9. Conclusions

The European EN 13201 series, established in 2016, addresses the rising concern of electricity costs for public lighting by establishing standards for street lighting designs. In Italy, the implementation of these standards, particularly EN 13201-1, through the national standard UNI 11248, introduced two dynamic dimming modes: TAI and FAI. While TAI relies solely on traffic flow data to dynamically adjust light intensity, FAI integrates additional inputs such as measured road surface luminance and meteorological data. Our study showcases the effectiveness of FAI through measurements carried out in real-world, FAI-enabled infrastructures in Vietnam and Italy, consisting of hundreds/thousands of streetlights. By comparing the measured energy consumption of FAI with that of traditional lighting-control methods, namely, virtual midnight, motion-sensing, and astronomical clock, we demonstrated the superiority of FAI. Specifically, field measurements show that FAI offers energy savings even larger than 50% compared to light controllers purely based on astronomical clocks. When compared with dynamic control strategies, such as virtual midnight and motion sensing, FAI proves its superiority as well, offering significant savings, especially on M3 and M4 streets, where regulations allow significant dimming in cases of low traffic.

## Figures and Tables

**Table 1:** Energy consumption over long periods of the reference 100W streetlight for M3, M4, and M5 streets.

	FAI	FAI without Rain	Virtual Midnight	Motion Sensing 1 min.	Motion Sensing 2 min.	Motion Sensing 3 min.	Motion Sensing 5 min.	Astronomical Clock
	[kWh]	[kWh]	[kWh]	[kWh]	[kWh]	[kWh]	[kWh]	[kWh]
	*Cesena (Italy). Observation period: 463 days between January 2023 and April 2024.*							
**M3**	240.7	208.2	447.0	415.0	440.9	457.3	484.5	549.4
	*Biassono (Italy). Observation period: 115 days between January and April 2024.*							
**M4**	65.5	62.4	111.4	121.5	123.6	124.9	128.6	135.9
	*Biassono (Italy). Observation period: 115 days between January and April 2024.*							
**M5**	113.2	110.4	129.2	138.6	139.5	140.0	142.1	146.1

**Table 2:** Comparison of energy savings relative to the astronomical clock for M3, M4, and M5 streets.

	FAI	FAI without Rain	Virtual Midnight	Motion Sensing 1 min.	Motion Sensing 2 min.	Motion Sensing 3 min.	Motion Sensing 5 min.
**M3**	56%	62%	19%	24%	20%	17%	12%
**M4**	52%	54%	18%	11%	9%	8%	5%
**M5**	22%	24%	11%	5%	5%	4%	3%

## Data Availability

The original contributions presented in the study are included in the article, further inquiries can be directed to the corresponding author.

## Abbreviations

The following abbreviations are used in this manuscript:

BLE	Bluetooth Low Energy
BMN	Bluetooth mesh networking
CLO	constant light output
ICT	information and communications technologies
IoT	Internet of Things
LoRa	long-range
LoRaWAN	LoRa wide area network
LC	ligh controller
LPWAN	low-power wide-area network
LTE	long-term evolution
MAC	medium access control
MF	maintenance factor
NB-IoT	narrowband-IoT
PAN	personal area network
PLC	power line communication
WAN	wide area network
WPAN	wireless personal area network
WSN	wireless sensor network
